# A mixed-methods feasibility study of an arts-based intervention for patients receiving maintenance haemodialysis

**DOI:** 10.1186/s12882-020-02162-4

**Published:** 2020-11-19

**Authors:** Claire Carswell, Joanne Reid, Ian Walsh, William Johnston, Helen McAneney, Robert Mullan, Jenny B. Lee, Hugh Nelson, Michael Matthews, Elizabeth Weatherup, Andrea Spencer, Jean Michelo, Anne Quail, Grainne Kielty, Alistair Mackenzie, Jenny Elliott, Nicola Arbuckle, Anna Wilson, Helen Noble

**Affiliations:** 1 School of Nursing and Midwifery, Queen’s University Belfast, Belfast, Northern Ireland UK; 2 School of Medicine, Dentistry and Biomedical Sciences, Queen’s University Belfast, Belfast, UK; 3 Kidney Care UK, Alton, UK; 4 Centre for Public Health, School of Medicine, Dentistry and Biomedical Sciences, Queen’s University Belfast, Belfast, UK; 5 Antrim Area Hospital, Northern Health and Social Care Trust, Antrim, UK; 6 College of the Arts, Center for Arts in Medicine, University of Florida, Gainesville, USA; 7 Northern Health and Social Care Trust, Antrim, UK; 8 Arts Care Northern Ireland, Belfast, UK; 9 Northern Ireland Kidney Patient Association, Belfast, UK; 10 South Eastern Health and Social Care Trust, Renal Unit, Belfast, UK

**Keywords:** Haemodialysis, Randomised controlled trials, Complex intervention, Arts in health, Arts-based intervention, End-stage kidney disease, Process evaluation, Feasibility study, Pilot study

## Abstract

**Background:**

Haemodialysis can negatively impact quality of life and mental health. Arts-based interventions used successfully in other settings to improve health and well-being, could help address the impact of haemodialysis. This study aimed to evaluate the feasibility and acceptability of conducting a randomised controlled trial (RCT) of an arts-based intervention for patients receiving haemodialysis.

**Methods:**

A parallel convergent mixed-methods design was used, including a pilot cluster RCT and qualitative process evaluation.

Phase 1 evaluated recruitment and retention rates through a pilot cluster RCT at a single haemodialysis unit in Northern Ireland. Participants included patients who received haemodialysis for ESKD, were over the age of 18 and had the capacity to consent. These participants were randomised to the intervention or control group according to their haemodialysis shift. The intervention involved six one-hour, one-to-one facilitated arts sessions during haemodialysis.

Phase 2 explored intervention and trial acceptability through a qualitative process evaluation using semi-structured interviews based on the RE-AIM framework. Participants included 13 patients who participated in phase 1 of the study, including 9 participants from the experimental group and four participants from the control group, and nine healthcare professionals who were present on the unit during implementation.

**Results:**

Out of 122 outpatient haemodialysis patients, 94 were assessed as eligible for participation. Twenty-four participants were randomised, meaning 80% of the target sample size was recruited and the attrition rate at 3 months was 12.5% (*n* = 3). Participants viewed the arts as more accessible and enjoyable than anticipated following implementation. All participants who started the intervention (*n* = 11) completed the full six sessions. Qualitative benefits of the intervention suggest improvements in mental well-being. Patient choice and facilitation were important factors for successful implementation.

**Conclusion:**

An arts-based intervention for patients receiving haemodialysis is acceptable for both patients and healthcare professionals, and a definitive trial is feasible. The intervention may help improve mental-wellbeing in patients receiving haemodialysis, but this requires further investigation in a definitive trial.

**Trial registration:**

The trial was prospectively registered on clinicaltrials.gov on 14/8/2018, registration number NCT03629496.

## Background

Haemodialysis is the most common form of dialysis worldwide [1] and is associated with a low health-related quality of life (HRQoL), high symptom burden and increased risk of anxiety and depression [2]. While haemodialysis is a life-sustaining treatment it can negatively impact mental health due to the psychological consequences of the treatment experience [3]. Mental health issues such as anxiety and depression also increase patients risk of morbidity and mortality, yet these issues are underdiagnosed and undertreated [4].

One component of the treatment experience thought to impact negatively on mental health and wellbeing is the issue of empty time during haemodialysis [3], where there is limited opportunity to engage in meaningful activities, creating profound boredom resulting in rumination and contemplation of illness and death [5]. Time can also be warped for patients during haemodialysis, as patients report time ‘dragging’ while watching the clock [3]. Arts-based interventions have been suggested in the literature as a vehicle to address this issue, as they provide an engaging activity during a difficult treatment, allowing distraction from existential boredom [6]. The arts also induce a subjective experience called a flow state, which can result in the perception of time passing much faster than it actually is [7]. Additionally, arts-based interventions have demonstrated some efficacy in reducing symptoms of anxiety and depression in other patient populations such as cancer, cardiovascular disease and diabetes [8].

However, there is a lack of evidence assessing their efficacy for improving health and wellbeing in patients receiving haemodialysis. The lack of randomised controlled trials (RCTs) of complex arts-based interventions for people with chronic illnesses is an established issue [9], compounded further by the challenges of conducting RCTs in nephrology. Trials in nephrology, the most under- researched field of internal medicine, are marked by problems with the retention of participants [10], partially because of the substantial treatment burden patients already experience and uncertainty around research processes [11]. Other issues that impact trials in nephrology include aspects of trial design, such as appropriate selection of outcome measures or practical aspects of trial conduct [12]. In order to address the issues that have historically impacted trials in nephrology, such as recruitment, attrition, randomisation and appropriate selection of outcome measures, it is important to conduct a feasibility study to identify issues that may impact a definitive trial, without jeopardising evaluations of efficacy.

Therefore, the aim of this study was to assess whether it is feasible to evaluate a complex arts-based intervention using this methodology before conducting a definitive trial.

## Methods

### Trial design

This study utilised a parallel convergent mixed method design including two phases; (i) a pilot cluster RCT, and (ii) a parallel qualitative process evaluation [13].

### Ethical approval and trial registration

Ethical approval was received from the Office of Research Ethics Northern Ireland and was prospectively registered on clinicaltrials.gov on 14/8/2018, registration number NCT03629496.

### Recruitment

#### Phase 1

A formal sample size calculation was not conducted as this is not appropriate for feasibility or pilot studies, as the objectives do not include hypothesis testing to establish the effectiveness of an intervention. The report of statistically significant results in pilots studies tends to be opportunistic, in that the study was not initially designed to establish effectiveness. This calls into question the validity of the effect, as the study was not designed for the purpose of hypothesis testing, and therefore would not have the rigour of a definitive RCT [14]. Feasibility studies should instead focus on the acceptability of trial processes or an intervention, including the willingness of participants to be randomised, the time needed to collect and analyse data and response rates to outcome measures [15–17].

There is little consensus on the appropriate sample size for a feasibility study, with guidance ranging from 12 per arm to 50 per arm [18]. The justification for larger sample sizes in feasibility studies is to obtain narrow standard deviations on outcome measures to maximise precision in a future power calculation [19]. However, whether this is an appropriate objective at the feasibility and piloting stage is debatable, as to obtain a narrow standard error for a precise power calculation the sample within the feasibility study would need to approach the size of a fully powered RCT [20]. This would in turn increase the likelihood of identifying a statistically significant effect during the feasibility stage, which can reduce the likelihood of a follow-up RCT [21]. A sample size of 30 is recommended by the NIHR’s Research Design Service for the estimation of a parameter, such as sample size, recruitment or attrition rate, for a definitive randomised controlled trial [22]. Due to the increased risk of identifying a statistically significant result with larger sample sizes, the objectives of the study, and the practical limitations of a small, single centre study, a sample size of 30 was selected for the pilot cluster RCT. A statistician at the Centre for Public Health at Queen’s University Belfast (HMcA), was consulted who confirmed this was an appropriate sample size to meet the objectives.

A convenience sample was used from an in-centre haemodialysis unit in Northern Ireland. Patients were screened and identified by clinical gatekeepers, and were approached by the primary researcher (CC) who explained the study in detail to the participant. Participants that expressed interest in participating were provided with a participant information sheet to take home and read. Patients were provided with a period of at least 48 h to decide if they wished to participate [23]. Those that agreed were provided with a consent form by CC. Reasons for ineligibility and for non-participation were captured in screening logs throughout the recruitment process. The recruitment process lasted approximately a month, from September to October 2018, during which time all eligible participants within the unit (*n* = 98) were approached and offered the chance to participate.

#### Phase 2

Participants for the process evaluation included both patients who had been recruited into phase 1 of the study, and healthcare professionals working on the haemodialysis unit during the implementation of the intervention.

Eligibility criteria for healthcare professionals:

- A member of the multidisciplinary team, including nurses, healthcare support workers, doctors, dietitians, social workers and counsellors.
- Have had experience with the intervention, meaning they had been present on the unit during implementation of at least one session of the intervention.
- Have worked in a clinical renal setting for more than 3 months.

During data collection for phase 1 patients were offered the opportunity to participate in the process evaluation. They were approached for participation once the implementation phase of the study had finished but before the three-month data collection time point. A purposive sampling strategy was used, with participants who would provide the richest data in the experimental group recruited. Participants in the control group were included to provide additional data on the trial processes and were purposively selected to provide a variety of experiences on random allocation and insight into the experience of the control condition.

Healthcare professionals were recruited for the process evaluation by purposive sampling. This sample technique was the most appropriate approach as it ensured the participants had the experience necessary to inform the research question. During the pilot cluster RCT the researcher was aware of what healthcare professionals were present during implementation of the intervention and used this to guide their sampling strategy. The ward manager acted as a gatekeeper and screened healthcare professionals to ensure that they met the inclusion criteria and sought permission for the researcher to approach them. Due to managerial and social hierarchies within healthcare, healthcare professionals may have felt pressure to participate when approached by their manager; therefore, the researcher provided the healthcare professionals with the participant information sheet and offered them a cooling-off period to consider participation, typically the time between the initial approach and their next shift on duty. This gave participants a minimum of 24 h to read through the information sheet and consider whether they wanted to participate in the process evaluation, in line with best practice [23]. Informed consent was then collected at the start of each interview.

### Randomization

Due to the highly interactive and collaborative nature of the intervention cluster randomisation was used to reduce the risk of contamination of control participants. All participants were informed that the study would involve random allocation to either engage in arts activities (experimental group) or be placed on a waiting list (control group). The initial randomisation procedure involved clustering participants according to the days of the week they attended haemodialysis [14]. However, due to differences in recruitment across these shift patterns, the clustering procedure was changed to ensure similar numbers in both intervention and control groups. Clusters were changed to the time of day participants attended for haemodialysis treatment.

The randomisation procedure was performed by a researcher not connected to the study. This involved flipping a coin that determined which group would receive the intervention, the allocation was placed in a sealed envelope which was then stored in a locked filing cabinet at the University site, along with the trial management file. The primary researcher (CC) opened the envelope once baseline data collection had been completed. Participants who attended the AM shifts were randomly allocated to the control group, and the participants who attended the PM shifts were randomly allocated to the intervention group.

### Intervention

The intervention was developed using the Arts in Health framework [24], guided by an interdisciplinary PPI advisory group [24], a scoping review [25], realist synthesis [6] and consultations with patients and healthcare professionals [26].

The intervention was developed based on the psychological theory of flow, which posits the existence of a ‘flow state’, a state of optimal experience that results from complete absorption in a task. The hallmark experience of a flow state is ‘tachypsychia’, an altered perception of time where typically time feels like it is passing faster than it actually is. In order to induce a flow state, the task must present a challenge to the individual that they can overcome through skill development [7]. Qualitative literature has suggested arts-in-medicine programmes can induce the hallmark experiences of a flow state in patients who participate, such as an altered perception of time and reduction in rumination and anxiety [26].

The intervention consisted of six one-hour, one-to-one facilitated arts sessions at the participant’s bedside during their haemodialysis treatment, and implemented by CC. The facilitator was a registered mental health and amateur artist who was involved in the development of the intervention. The development of the intervention, and intervention manual, have been described elsewhere [26]. These sessions happened twice a week for a period of 3 weeks for each participant. Participants had a choice between creative writing or visual art during each session, as these could be safely implemented within the clinical constraints of haemodialysis treatment, and participants could choose from a selection of art materials and prompts.

Adherence, fidelity and dose of the intervention was monitored using activity logs completed by the facilitator, documenting the time spent implementing each art session, the activities the participants engaged in, any instances of non-participation and reasons, general feedback from patients during the sessions, instances of contamination between the control and experimental group and any adverse effects experienced by participants.

### Control

Participants from the control group were asked not to participate in any active arts activities during their haemodialysis sessions throughout the study, but that once data collection was completed they would have an opportunity to take part in art sessions and receive a pack of art supplies. This was not a form of delayed entry as no data was collected from the control group once the follow-up time period had finished. The provision of the arts pack following the study was recommended by the interdisciplinary advisory group to promote retention of participants, and the waiting list design was recommended by the OREC NI ethics panel to ensure all participants had the opportunity to receive guidance and instruction on how to use the arts materials.

### Outcomes

#### Phase 1 – feasibility measures

The main feasibility outcome of interest was the recruitment rate of participants. Assessing the ability to recruit participants is a common issue explored in feasibility trials [17, 18, 27]. Screening, approach and recruitment logs were kept during the recruitment phase of the study to capture the proportion of patients eligible and who consented to participation, and reasons for non-participation or ineligibility.

Nephrology and research involving patients with end-stage diseases experience high attrition rates having a detrimental impact on the quality of evidence available in these fields [27]. Therefore, the ability to retain participants is an important consideration prior to a definitive RCT. The attrition rate of participants over a three-month period (from baseline to final follow-up) was captured during data collection and intervention implementation. Reasons for withdrawal were documented in participant’s case report forms (CRF) to identify any modifiable factors that contributed to attrition.

Other outcomes captured during phase 1 included the acceptability of randomisation and clustering method according to differences in demographics and attrition rates between groups; acceptability, adherence and fidelity of the arts-based intervention according to adherence, completion rate and time participants engaged in arts sessions as recorded in the activity logs, and acceptability of clinical outcome measures according to completion rates and proportion of missing data.

#### Phase 1 – clinical outcome measures

Clinical outcome measures were administered to explore the acceptability and appropriateness of these outcomes within a definitive trial, as opposed to evaluating the effectiveness of the intervention. Baseline demographic and clinical data included age, gender, ethnicity, education, dialysis vintage, dialysis access, frailty as measured by the clinical frailty scale and number of self-reported co-morbidities [28]. These data were collected to explore potential demographic barriers to participation and to explore differences between groups to evaluate the acceptability of the clustering procedure in a definitive trial.

Arts-based interventions can improve depression and have also been found to improve QoL in observational studies of patients receiving haemodialysis. Therefore the Kidney Disease Quality of Life Short Form 36 (KDQoL-SF36) [29], and the Hospital Anxiety and Depression Scale (HADS) [30] were administered at baseline, immediately post-intervention, at six-week and three-month follow-up. Arts-based intervention research has faced criticism due to lack of longitudinal follow-up, which would be necessary to identify whether any benefits are sustained following cessation of the intervention [9]. Participants who are lost to follow-up in longitudinal RCTs concerning complex healthcare interventions tend to be older, diagnosed with a chronic illness and have higher levels of co-morbidity [31], common demographic factors in patients with ESKD [32]. To establish the feasibility of follow-up within a definitive RCT and establish attrition rates over a longer period of time, participants were follow up at 6-weeks and 3-months post-baseline.

#### Phase 2 – qualitative process evaluation

This phase of the study explored acceptability of the arts-based intervention and the trial processes for both patients and HCPs using semi-structured interviews. The semi-structured interviews used interview guides consisting of open questions informed by the RE-AIM QuEST framework [33]. The RE-AIM framework outlines that the reach, effectiveness, adoption, implementation and maintenance of an intervention should be explored with both qualitative and quantitative measures, in order to identify any necessary modifications to improve future implementation, both for replication in research and translation into clinical practice [33, 34]. The interview guides used open ended questions to ensure participants could express and explore perspectives that they considered relevant to implementation, but the questions themselves were targeted and specific to the RE-AIM framework to ensure the responses were relevant to informing a larger trial.

Semi-structured interviews were conducted with nine participants from the intervention group, four from the control group, and nine HCPs who had experienced the intervention. Interviews were conducted until data saturation was reached across all three subgroups of participants. The interviews with HCPs and participants from the control group were conducted by CC, whilst interviews with participants from the intervention were conducted by HN and CMcK to reduce bias.

### Statistical analysis

#### Quantitative data

Data analysis was conducted using the Statistical Package for the Social Sciences (SPSS v 24). Descriptive statistics were used to present baseline demographic and clinical data. Categorical data was presented as frequencies and percentages, while continuous data was presented as means and standard deviations. Recruitment, participation and retention rates were reported and presented in a CONSORT flow diagram [14]. Exploratory inferential statistics were conducted, but no conclusions on the effectiveness of the intervention were made from the results and therefore these were not reported.

#### Qualitative data

The semi-structured interviews were recorded and transcribed verbatim, and data were managed using NVivo Version 11. Inductive thematic analysis was used to identify overarching themes and sub-themes relating to the objectives of the process evaluation [35] and the RE-AIM framework [33, 34]. The transcripts and identified themes were reviewed, revised and finalised collaboratively by CC, HN, JR and IW.

### Progression criteria

Progression to a definitive RCT was determined by recruitment rates and the acceptability of the intervention [10]. The progression criteria for the primary outcomes of recruitment and attrition were based on similar feasibility studies on arts-based interventions in other clinical populations [36] and were confirmed as suitable for the objectives of the study by the statistician at the Centre for Public Health at Queen’s University Belfast (HMcA):

- 75–100% of the target sample size recruited from a single site would result in progression to a definitive RCT
- More than 20% attrition rate from the recruited sample will result in revision of the protocol and data from the process evaluation, and appropriate amendments will need to be made to address barriers to retention of participants, prior to progression to a full trial [37].

### Integration of quantitative and qualitative results

The results were integrated following analysis in order to adequately address the objectives and over all aim of the study. This was achieved by constructing a table that displayed the main quantitative and qualitative findings relating to the key objectives. Quantitative findings were presented and supporting and contradicting qualitative data were identified [38]. Where appropriate other quantitative data was also provided to support or contradict a finding. Whilst the quantitative data were used as a reference point for comparison this was not a consequence of the primacy of the quantitative data, instead the qualitative data provided more nuanced and complex insights that enabled the identification of supportive and contradicting points.

## Results

### Phase 1

#### Recruitment

**Fig. 1:**
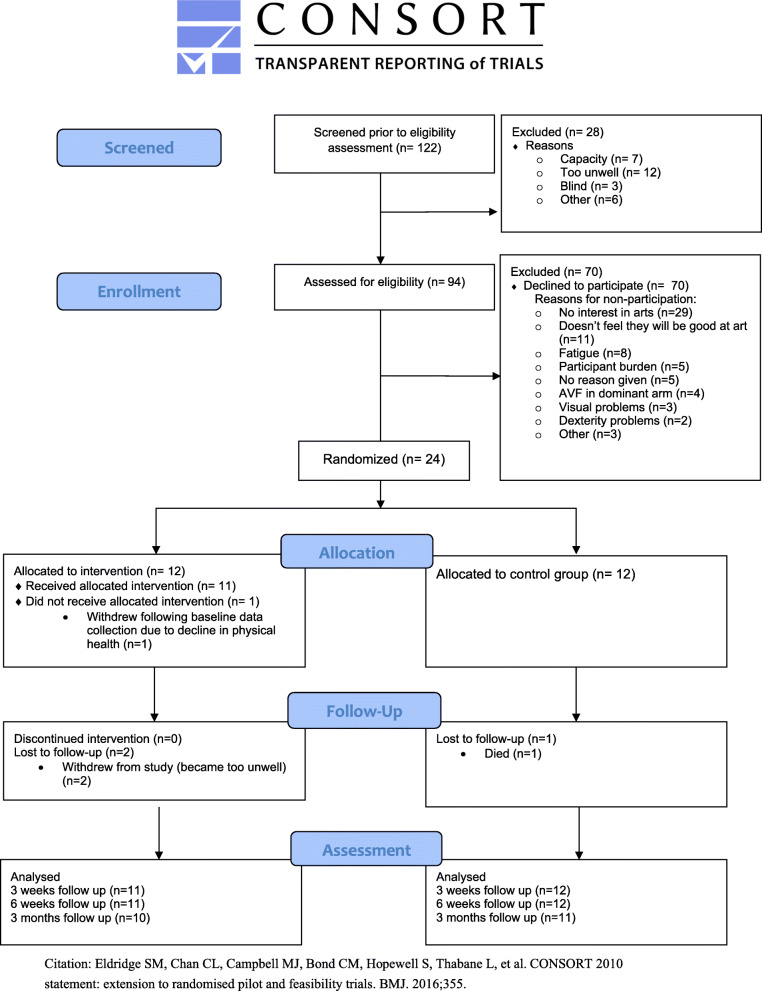
CONSORT flow diagram

Out of 122 outpatient haemodialysis patients, 94 were assessed as eligible for participation. Seventy participants declined to participate (74.5% of eligible participants). The most common reasons being a lack of interest in the arts (*n* = 29), and anxiety related to a perceived lack of creative ability (*n* = 11). Twenty-four participants (25.5% of eligible participants) consented to participate in the study and were successfully randomised to control or intervention group. Therefore 80% of the target sample size was successfully recruited to the study. An overview of screening, recruitment and attrition can be seen in the Consort flow diagram in Fig. 1. The demographics of recruited participants at baseline are presented in Table 1.

**Table 1:** Baseline demographic and clinical characteristics of participants

	Intervention group (*n* = 12)	Control group (*n* = 12)	Total (*n* = 24)
Age, years, mean (SD*)	71 (13.03)	66.08 (15.72)	68.54 (14.34)
Range	37–82	40–88	37–88
Marital status n (%)			
• Married	5 (41.7%)	8 (66.7%)	13 (54.2%)
• Widowed	2 (16.7%)	2 (16.7%)	4 (16.7%)
• Single	5 (41.7%)	2 (16.7%)	7 (29.2%)
Gender n (%)			
• Male	5 (41.7%)	7 (58.3%)	12 (50%)
• Female	7 (58.3%)	5 (41.7%)	12 (50%)
Vascular access n (%)			
• Arteriovenous fistula dominant arm	1 (8.3%)	0	1 (4.2%)
• Arteriovenous fistula non-dominant arm	4 (33.3%)	4 (33.3%)	8 (33.3%)
• Central venous catheter	7 (58.3%)	8 (66.7%)	15 (62.5%)
Level of Education n (%)			
• Primary School	1 (8.3%)	2 (16.7%)	3 (12.5%)
• Secondary School	11 (91.7%)	10 (83.3%)	21 (87.5%)
• Higher education	0	0	0
Ethnicity n (%)			
• White	12 (100%)	11 (91.7%)	23 (95.8%)
• African	0	1 (8.3%)	1 (4.2%)
Clinical frailty n (%)			
• Well	0	1 (8.3%)	1 (4.2%)
• Managing well	2 (16.7%)	0	2 (8.3%)
• Vulnerable	1 (8.3%)	1 (8.3%)	2 (8.3%)
• Mildly frail	3 (25%)	5 (41.7%)	8 (33.3%)
• Moderately frail	2 (16.7)	5 (41.7%)	7 (29.2%)
• Severely frail	4 (33.3%)	0	4 (16.7%)
Dialysis vintage, years, mean (SD)	3.23 (3.96)	5.31 (4.37)	4.28 (4.21)
Range	.08–14	.25–14	.08–14
Number of co-morbidities, mean (SD)	2.08 (2.2)	3.67 (2.96)	2.88 (2.68)
Range	0–6	0–12	0–12

*SD* Standard deviation

#### Retention

There was an overall attrition rate of 12.5% (*n* = 3) during the study. Two participants withdrew from the intervention group and one from the control group. Reasons for withdrawal centred around physical health; one participant withdrew from the study following baseline data collection as they sustained injuries that made them physically unable to participate in the intervention, one participant withdrew at three-month follow-up due to a significant decline in their physical health, and one participant died shortly before the three-month data collection time point. No participants withdrew from the study whilst engaging with the intervention and no participants withdrew from the study because of participant burden.

#### Intervention adherence and fidelity

One participant withdrew from the study prior to starting the intervention. All 11 participants who started the intervention completed six art sessions over the course of 3 weeks. As there were only two arts sessions per participant each week, this provided flexibility to allow participants to rearrange their sessions if required.

While it was planned that sessions would be limited to an hour, during implementation it became apparent that this restriction was limiting engagement. As participants developed skills their engagement increased and they required more time and to complete their work. Consequently, the mean length of time increased for each participant over the course of the six sessions, with the initial session lasting hour (58.8 min, SD = .9) and the mean length of the final session lasting approximately 69.6 min (SD = .16.2).

#### Clinical outcome measures

The majority of participants completed the questionnaires with assistance of the researcher (CC), only two participants completed the questionnaires without assistance. Using this approach there was no missing data across all outcome measures at each time point, with the exception of participants who withdrew from the study.

Table 2 shows the mean, standard deviation and 95% confidence intervals of the clinical outcome measures at baseline for all participants. The mean HADS score for both anxiety and depression were lower in the intervention group (3.42 and 2.75 respectively) when compared to the control group (8.33 and 7.33). This suggests that the two groups were not similar at baseline in terms of their levels of anxiety and depression. The mean scores across the different KDQOL-SF 36 subscales were also consistently higher in the intervention group compared to the control group, suggesting the two groups were not similar at baseline in terms of their HRQoL.

**Table 2:** Baseline clinical outcome measures

Baseline Outcome Measures									
Clinical outcome measure	All participants (*n* = 24)			Intervention group (*n* = 12)			Control group (*n* = 12)		
	Mean (SD)	Median (IQR)*	95% CI	Mean (SD)	Median (IQR)*	95% CI	Mean (SD)	Median (IQR)*	95% CI
HADS Anxiety	5.88 (5.53)	4 (6.5)	3.54–8.21	3.42 (3.32)		1.31–5.52	8.33 (6.31)	5.50 (12)	4.32–12.35
HADS Depression	5.05 (5.68)	3 (7.75)	2.64–7.44	2.75 (2.86)		.93–4.57	7.33 (6.92)		2.94–11.73
KDQOL Symptom list	70.76 (22.09)		61.43–80.08	78.82 (21.28)		65.30–92.34	62.69 (20.63)		49.58–75.80
KDQOL Effects of kidney disease	66.19 (28.60)	72.5 (50.78)	54.12–78.27	76.29 (22.51)		61.99–90.59	56.10 (31.32)		36.19–75-99
KDQOL Burden of kidney disease	33.85 (29.37)	31.25 (35.94)	21.45–46.26	43.23 (29.85)	34.38 (34.38)	24.26–62.20	24.48 (26.84)	18.95 (29.69)	7.42–41.53
KDQOL Physical composite	29.99 (11.49)	27.38 (13.16)	25.14–34.84	34.31 (13.33)	30.46 (25.33)	25.76–42.70	25.75 (7.70)		20.86–30.64
KDQOL Mental composite	49 (13. 70)	51.52 (17.32)	43.22–54.78	55.03 (6.98)		50.60–59.47	42.97 (16.25)		32.64–53.30
EQ-5D-5L VAS	49.71 (24.29)		39.45–59.96	57.92 (23.76)		42.82–73.01	41.50 (22.84)		26.99–56.01

*Median and interquartile range presented due to statistically significant result on Shapiro-Wilk test (*p* ≤ .05)

**Fig. 2:**
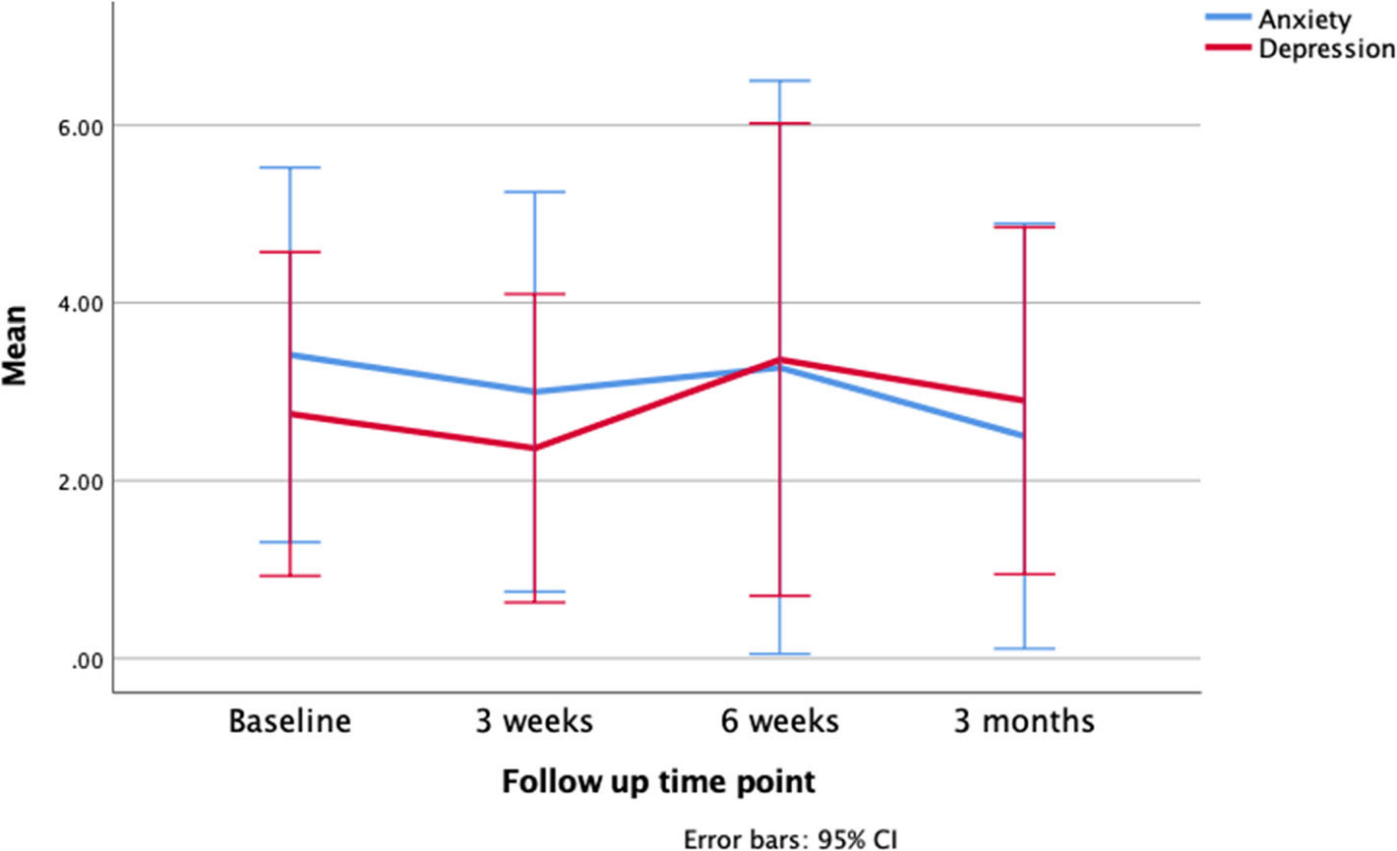
Mean anxiety and depression scores for the intervention group over 3-month longitudinal follow-up

**Fig. 3:**
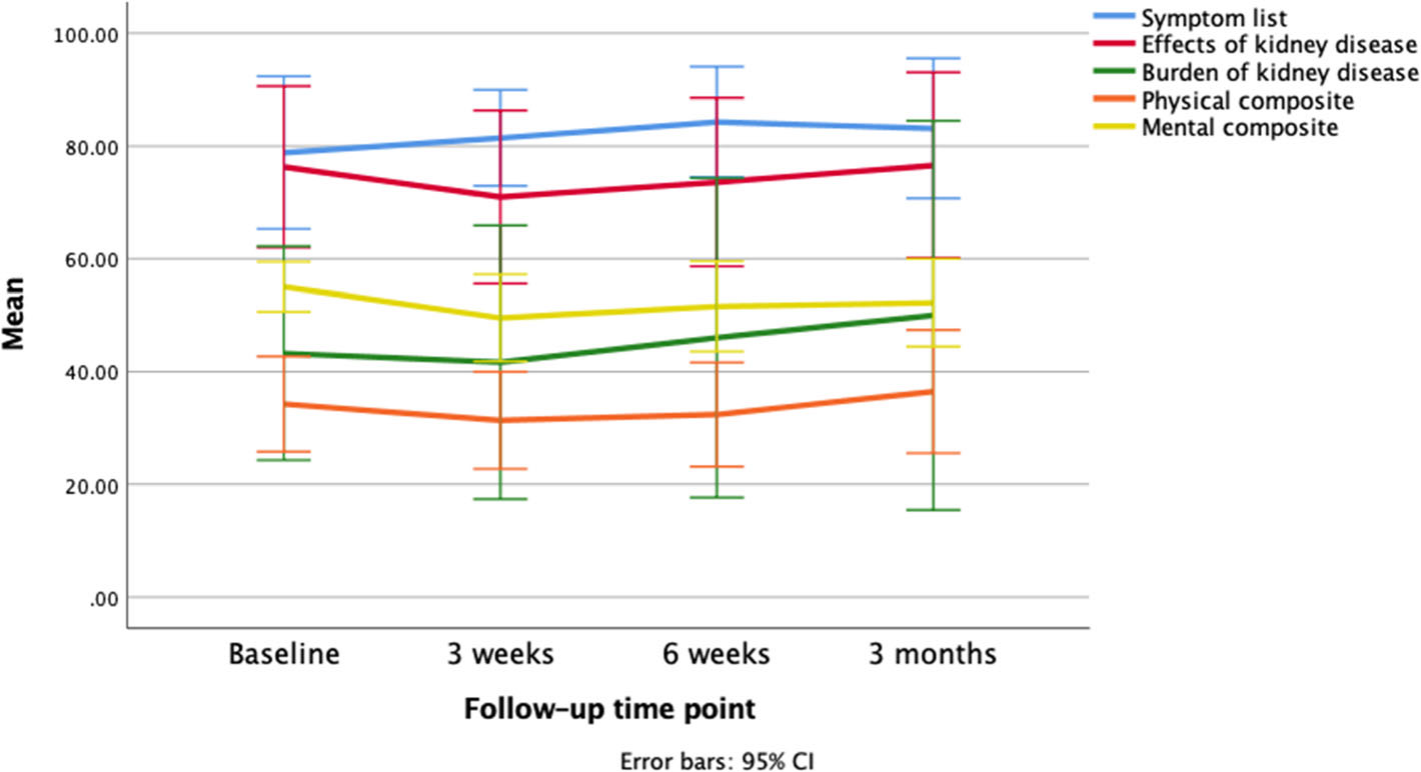
Mean KDQOL-SF 36 subscale scores f or the intervention group over 3-month longitudinal follow-up

Figure 2 shows the mean anxiety and depression scores for the intervention group over 3-months longitudinal follow-up. A similar reduction in anxiety and depression was also found in the control group over the three-month follow-up time period. The reduction in anxiety and depression is more pronounced in this group as they had notably higher mean anxiety and depression scores at baseline when compared to the intervention group. Figure 3 shows the mean KDQOL-SF 36 subscale scores for the intervention group over 3-months longitudinal follow-up, showing a general trend of mean increases at 6 weeks and 3 months follow-up, after the initial apparently inconsistent changes across subscales in the post-intervention period. The control group also demonstrated varied changes across different subscales of the KDQOL-SF 36 during the longitudinal follow-up period, but at 3 months all subscales had improved compared to the initial baseline mean scores.

### Phase 2

An overview of interviewee characteristics are provided in Table 3. Nine participants were recruited from the intervention group, four from the control group, and nine healthcare professionals were recruited who had observed implementation of the intervention whilst working on the unit.

**Table 3:** Interviewee characteristics

Characteristic	No. of participants
**Intervention group**	
Male	5
Female	4
**Total**	**9**
**Control group**	
Male	2
Female	2
**Total**	**4**
**Healthcare professional**	
Renal nurse (Band 5)	6
Ward manager	1
Healthcare support worker	2
**Total:**	**9**
**All interviews**	
Male	7
Female	15
**Sub-total:**	**22**

A total of four themes and 17 subthemes were identified through the semi-structured interviews. An overview of the themes, subthemes, and the participants who contributed to the identification of these themes are provided in Table 4. Data will be presented to outline each theme in turn.

**Table 4:** Process evaluation themes and sub-themes

Themes	Subthemes	Identified by	Supporting quotation
Perception of art	Mixed preconceptions and apprehensions of art participation	Experimental group, control group and healthcare professionals	*My first thought of it [the arts-based intervention] was like how would this even be possible with some patients with only one arm, um, able to do stuff.*
	Improved perception of art participation following intervention	Experimental group and healthcare professionals	*I was coming from a place where I wasn’t interested in art, I didn’t think I could do it, and she sort of…. How would I put it? Encouraged me, trailed me through it.*
	Negative appraisal of abilities	Experimental group and control group	*Half pleased with it, and half not. I didn’t like the way I got the apples, I was trying to make them red and green, but it just didn’t turn out the way I want it.*
Effect of the arts-based intervention	Generation of positive affect due to exposure to arts-based intervention	Experimental group and healthcare professionals	*I think they really enjoyed it, like, you know, they definitely – it was great, I would love to see it in the unit. You know the patients… you get a different side to the patients, and they were so much happier, I think.*
	Improved self-esteem	Experimental group, control group and healthcare professionals	*Oh, well, I suppose satisfaction that you can add another string to your bow at 80 odd years of age.*
	Sense of purpose	Experimental group, control group and healthcare professionals	*Well, it gave me something to go for, like a goal… gave me a goal to go for, you know… You go for that and you keep going for it, you know.*
	Increased social interaction	Experimental group and healthcare professionals	*So they were getting that one-to-one time where it was something that was about them and it was positive rather than them being sick and me standing over them. So I thought it was great*
	Positive impact of the arts-based intervention on the dialysis experience	Experimental group and healthcare professionals	*It made it look like we just didn’t see it as a dialysis environment, that we were actually looking at different aspects of the patient’s wellbeing.*
Acceptability of the arts-based intervention	Adaptation of the arts-based intervention to the constraints of a haemodialysis setting	Experimental group and healthcare professionals	*See my hand’s tied down. I wouldn’t be able to paint with my right hand, that means I had to paint with my left hand.*
	Positive influence of facilitator on participant experience	Experimental group and healthcare professionals	*She says ‘Well try (participants name)’ She says ‘it’ll be your own painting, it’ll not be the same, nobody’s two paintings are the same. ‘Cause that’s your painting, and that other ones somebody else’s, what they say, just paint what you see… Yes, it made me feel good because she thought it was good and she was learning me.*
	Importance of participant choice on subject and activity within the arts-based intervention	Experimental group and healthcare professionals	*It was supposed to be a scene of you know, eh, by the beach, a beach and water and clouds. Awful! Blob. Blob. Blob. So the next time [the facilitator] arrived she said ‘would you try drawing?’… And I said ‘that’s going to be worse’. So she handed me this pencil, she worked out what was a good pencil for giving a result, and when I started she said ‘you can draw’*
	Length of the arts-based intervention	Experimental group and healthcare professionals	*But some days I thought it was pretty short and other days… well it wasn’t too long, it was never too long.*
	Quality and suitability of materials for the arts-based intervention	Experimental group, control group and healthcare professionals	*It was all… catered to the environment. It was fit for the environment. Even if you used water based, they were not like messy water based… what children would use, you know. So it was all very… very accurate. Very well done.*
Acceptability of research procedures	Factors influencing recruitment	Experimental group and control group	*But I do think if they’d actually known what was gonna happen, you know, like pure… I would say they probably just seen paperwork and they went – a lot of them we’re doing like kitchen questionnaires with them, like… you know what I mean?*
	Completion and acceptability of outcome measures	Experimental group and control group	*I answered the question, but I couldn’t see it’s got anything to do with me on dialysis and doing art.*
	Acceptability of randomisation	Experimental group and control group	*Maybe if I had have seen them happening in the ward, maybe it would have annoyed me because I maybe would have wanted to join in, especially if there was laughter and all because then of course, you think you’re missing something.*
	Retention of participants	Experimental group, control group and healthcare professionals	*Well there was no point in starting it if you were going to walk away from it!*

#### Theme 1: perception of art

This theme highlights the interviewee’s perception of art participation, both prior to the intervention, during and following implementation. Participants had mixed preconceptions and apprehensions of art, with patients saying they were ‘*a bit nervous’* mostly due to a lack of experience, and were consistently critical of their artistic skills.

*The first one was absolutely dreadful! Because it was painting, and I just ended up with this… blobs all over the place. It was supposed to be a scene of you know, eh, by the beach, a beach and water and clouds. Awful! Blob. Blob. Blob.*

*-EG06*

However, participants’ reported a change in their perception of art participation following implementation of the intervention. The changes in perception were exclusively positive, with participants identifying an interest where they had none previously.

*I was quite happy I done it and I was quite happy with the results… You know, and it gives you something – you achieve something,*

*-EG01*

#### Theme 2: effect of the arts-based intervention on patients and staff

This theme captured the effects the intervention had on both patients and HCPs. The art triggered positive emotional responses amongst participants, with improvements in self-esteem and the development of a sense of purpose being commonly reported as it helped participants ‘*achieve something’*.

*‘I suppose satisfaction that you can add another string to your bow at 80 odd years of age.’*

*-EG03*

There was an increase in social interaction both between patients and between patients and HCPs as ‘*it gave them a bond’*, and improved the dialysis experience for both patients and HCPs. Participants reported that the intervention required a degree of focus and concentration, and consequently they were distracted from both their thoughts and their surroundings, improving their intradialytic experience and altering patients’ perception of time in a beneficial way, by seemingly making the time pass quicker as they were focused on a task and no longer watching the clock.

*Aye the time seemed to go in a bit quicker whenever … comes in, you watch TV but uh, you never notice the time so much. But whenever you’re painting time, that hour or whatever it was that she was in with, just seemed to go like that.*

*– EG05*

Healthcare professionals reported that the intervention had an impact on the environment itself by transforming the atmosphere into one where the focus wasn’t exclusively clinical:

*This was sort of taking it to the next level and saying ‘it’s not all about dialysis, it’s not all about kidneys here, we’re looking after you and we are seeing what can benefit you positively here’*

*– HCP04*

#### Theme 3: acceptability of the arts-based intervention

This theme explored the acceptability of the intervention within the haemodialysis setting for both patients and HCPs. There are six sub-themes related to the intervention’s acceptability, addressing the intervention, implementation strategies and context (Table 4). Participants identified potential barriers to implementation related to the haemodialysis setting, but highlighted how these barriers were easily overcome.

*Not even patients with fistulas! Did that affect them? No! (Laughs) Not at all! So, if you can get over that barrier and they’re happy – yeah, there were no problems.*

*-HCP09*

The presence of a facilitator was identified as beneficial, both for overcoming barriers and producing a positive experience, as they were perceived as ‘*a good teacher’*. Participants highlighted the importance of choice and variety for sustained engagement ‘*because they weren’t Pidgeon-holed’*, as well as the suitability of materials for the haemodialysis environment.

*It was all… catered to the environment. It was fit for the environment. Even if you used water based, they were not like messy water based… what children would use, you know. So it was all very… very accurate. Very well done.*

*-HP06*

#### Theme 4: acceptability of research procedures

This theme highlighted the acceptability of trial design and procedures through four sub-themes. Participants identified curiosity and boredom as main motivating factors for participation.

Clustering was identified as essential for the acceptability of randomisation, as ‘*it would have annoyed’* control participants to view others engaging in the intervention, whilst a waiting list was important to reduce attrition in the control group as participants reported that this as a core motivation for remaining in the study.

*Just looking forward to it… to doing the art, just something different to amuse me here. Instead of lying sleeping.*

*-CG03*

The outcome measures were not perceived as burdensome to participants however they weren’t seen as appropriate for capturing the benefits of the arts.

*I answered the question, but I couldn’t see it’s got anything to do with me on dialysis and doing art.*

*-EG03*

## Discussion

This is the first feasibility study to pilot a cluster RCT of a complex arts-based intervention for patients receiving haemodialysis, and establish the acceptability of the intervention for patients and healthcare professionals. 80% of the target sample was successfully recruited from a single site, showing recruitment to a definitive trial is likely feasible. Reasons for non-participation included lack of interest in art and anxiety over perceived dearth of artistic ability. Lack of interest is a common reason for non-participation in complex intervention trials for patients receiving haemodialysis, and is not specific to arts-based interventions [39]. The recruited sample is also similar to other pilot studies evaluating intradialytic interventions [40]. Additionally, the overall recruitment rate for this study (24 participants at a single site over 1 month) is notably higher than the median recruitment rate for NIHR funded trials in general (0.92 participants at a single site over 1 month) [41], however the unique nature of haemodialysis treatment makes it difficult to draw comparisons with other patient populations who attend hospital at a much lower frequency.

The process evaluation provided potential strategies for improving recruitment, suggesting that these barriers may be modifiable. Suggested strategies included demonstration of the intervention at the bedside during recruitment, or inclusion of examples of completed artistic work included in the participant information sheets. Feasibility studies of arts-based interventions in other patient groups have reported relatively low recruitment rates in terms of the proportion of eligible patients successfully recruited, with a recent feasibility study exploring a community arts-based intervention for patients who have experienced stroke reporting a recruitment rate of 14% (56/392) [42]. However, in comparison our proportional recruitment rate of 25.5% (24/94) was notably higher, suggesting that there are contextually different factors that facilitate recruitment in haemodialysis settings.

There was an attrition rate of 12.5%, with three participants withdrawing during the study, suggesting retention of participants in a definitive trial is feasible. Participants were withdrawn from the study because of significant declines in their physical health (*n* = 2) and death (*n* = 1), reflecting the high level of frailty and mortality in this population [32]. There will likely be similar mortality and frailty issues in a definitive trial and this should be accounted for in the sample size calculation and the length of longitudinal follow-up. One crucial finding was that participants in the control group did not withdraw as a result of participant burden, but instead remained within the study as a result of the waiting list control design. Participants were willing to wait to receive an intervention that most had no experience with, because they would have the opportunity to do something during haemodialysis. This emphasises just how prevalent the problem of boredom is for patients receiving haemodialysis [3], and additionally that the desire to use this time productively can act as a facilitator for participant recruitment and retention in haemodialysis settings.

Other aspects of the trial processes were also determined to be feasible with slight modifications, including cluster randomisation and administration of outcome measures. While the clustering method was acceptable to patients and resulted in a similar number of participants in each arm, the notable differences in demographics, anxiety and depression demonstrated that the clusters were not similar at baseline. Previous research has demonstrated that patients who attend for haemodialysis in the morning have significantly higher rates of anxiety and depression, especially when compared to patients attending haemodialysis in the evening [43, 44]. Therefore, trials conducted in nephrology should account for the impact that shift patterns may have on important patient outcomes.

The process evaluation revealed that participants did not feel the outcome measures were appropriate for capturing the benefits of the intervention, and therefore a more appropriate primary outcome measure needs to be identified before the trial is scaled up. This is supported by the quantitative results of the KDQOL-SF 36 which demonstrated inconsistent changes across all subscales throughout the study for both intervention and control groups, likely as a result of random variation. There is a focus in nephrology research on HRQoL outcomes that emphasise the importance of physical health and function, as opposed to mental health and wellbeing [45]. It is likely that measures focused on HRQoL in kidney disease are not best placed to measure the impact of interventions that aim to address psychological, social and emotional issues that patients experience. While the pattern of change in anxiety and depression in the intervention group suggests it may be an appropriate outcome in a definitive trial, as anxiety and depression reduced post-intervention, there is an additional concern that the symptoms of these conditions overlap with symptoms of kidney disease [46]. Having outcomes that focus on psychological aspects of these conditions, or positively phrased outcomes that do not focus on the presence or absence of symptoms, may address this problem in future research.

Acceptability of the intervention was highlighted throughout the process evaluation, both for patients and HCPs. Whilst participants identified potential barriers to implementation, these had previously been identified during the development of the intervention [6, 23] and therefore were resolved prior to implementation. The intervention was not burdensome or disruptive for HCPs, an issue that can negatively impact clinical care [47]. This feasibility study demonstrated that by placing patients and HCPs at the centre of intervention development, particularly one designed to be intradialytic, can help reduce the impact of the intervention on the adequacy and safety of the patients treatment, and even reduce burden placed on HCPs in the setting.

Instead the intervention provided benefits for both patients and HCPs, potentially by inducing flow states amongst patients [48], promoting mental wellbeing amongst patients and HCPs [49] and transforming the atmosphere of the clinical environment [50]. Whilst patients did report experiences that would indicate they were experiencing flow states while engaging in the intervention, art appeared to provide a more holistic impact on their wellbeing by improving their self-esteem, providing participants with a sense of purpose, promoting social engagement and generating positive affective states. Consequently, the experiential benefits of the arts-based intervention more accurately address the core components of Seligman’s PERMA model of wellbeing [49]. Patients with kidney disease have lower levels of mental wellbeing compared to the general population [51], and this is associated with higher levels of mental illness. However, mental wellbeing is a topic that is rarely explored in nephrology, in part due to the focus on physical health outcomes [52]. Due to the association between mental wellbeing, mental morbidity and the impact this can have on treatment and medication adherence, this outcome deserves further exploration in this population [51].

### Strengths and limitations

This study enabled issues of trial and intervention feasibility to be explored without jeopardising the validity of a definitive trial, and identified modifiable issues that will improve the rigour of a future RCT. The mixed methods approach enabled aspects of the trial and intervention to be explored in-depth and strategies developed to improve future research methodology. A limitation of this study was the lack of differentiation of roles within the research team, due to resource limitations. Consequently recruitment, facilitation of the intervention, and quantitative data collection was conducted by the primary researcher (CC). The established relationship between the researcher/facilitator and participants could have improved retention and completion of outcome measures. However, the control group also had a high response rate and low attrition rate despite having notably less engagement with the researcher, implying this may not be an issue in a definitive trial.

## Conclusions

This study has implemented a complex arts-based intervention that is both safe and acceptable for patients receiving haemodialysis and HCPs, and has demonstrated that it is feasible to evaluate the effectiveness of this intervention within a definitive cluster RCT with some small modifications. The definitive study should consider the impact of these complex interventions outside the target patient group, for example, on HCPs working on the unit where implementation is taking place. This study demonstrated that there are numerous facilitators to RCTs within the haemodialysis setting, and identified potential avenues for improving the quality of trials in nephrology, particularly for intradialytic interventions.

## Data Availability

Due to the size and nature of the data set participants may be identifiable from the raw data. The datasets analysed during the current study are available from the corresponding author on reasonable request.

## Abbreviations

HCP: Healthcare professional
HRQoL: Health related quality of life
MRC: Medical Research Council
NHS: National Health Service
NICE: National Institute for Health and Care Excellence
NIHR: National Institute for Health Research
QoL: Quality of life
RCT: Randomised Controlled Trial
